# Comparison of time to negative conversion of SARS-CoV-2 between young and elderly among asymptomatic and mild COVID-19 patients: a cohort study from a national containment center

**DOI:** 10.3389/fmed.2024.1217849

**Published:** 2024-03-18

**Authors:** Imen Zemni, Cyrine Bennasrallah, Ines Charrada, Wafa Dhouib, Amani Maatouk, Donia Ben Hassine, Rim Klii, Meriem Kacem, Manel Ben Fredj, Hela Abroug, Salma Mhalla, Maha Mastouri, Chawki Loussaief, Ines Jlassi, Ines Bouanène, Asma Sriha Belguith

**Affiliations:** ^1^Department of Epidemiology and Preventive Medicine, Fattouma Bourguiba University Hospital, University of Monastir, Monastir, Tunisia; ^2^Faculty of Medicine of Monastir, Department of Epidemiology, University of Monastir, Monastir, Tunisia; ^3^Technology and Medical Imaging Research Laboratory, University of Monastir, Monastir, Tunisia; ^4^Department of Endocrinology, Fattouma Bourguiba University Hospital, University of Monastir, Monastir, Tunisia; ^5^Department of Microbiology, Fattouma Bourguiba University Hospital, University of Monastir, Monastir, Tunisia; ^6^Department of Internal Medicine, Fattouma Bourguiba University Hospital, University of Monastir, Monastir, Tunisia; ^7^Department of Infectiology, Fattouma Bourguiba University Hospital, University of Monastir, Monastir, Tunisia; ^8^Faculty of Sciences of Monastir, Department of Mathematics and Statistics, University of Monastir, Monastir, Tunisia

**Keywords:** COVID-19, elderly, pandemic, viral clearance, mild COVID-19 infection

## Abstract

**Objective:**

We aimed to study the relationship between age and time to negative conversion of SARS-CoV-2 in patients with asymptomatic and mild forms of COVID-19.

**Methods:**

We conducted a cohort study including all patients diagnosed with COVID-19 from the national COVID-19 containment center of Tunisia. Patients were subdivided into two cohorts: (under 60 years) and (over 60 years) and were followed up until PCR negativization. Log rank test and Cox regression were applied to compare time to negative conversion between the old group and the young group.

**Results:**

The study included 289 patients with non-severe forms of COVID-19. Age over 60 was significantly associated with delayed negative conversion in male sex (Hazard ratio (HR): 1.9; 95% CI: 1.2–3.07) and among patients with morbid conditions (HR:1.68; 95% CI: 1.02–2.75) especially diabetics (HR: 2.06; 95% CI: 1.01–4.21). This association increased to (HR:2.3; 95% CI: 1.13–4.66) when male sex and comorbidities were concomitantly present and rose to (HR: 2.63; 95% CI: 1.02–6.80) for men with diabetes. Cox regression analysis revealed a significantly delayed negative conversion in symptomatic patients. Significant interaction was observed between gender and age and between age and chronic conditions.

**Conclusion:**

Age is associated with delayed negative conversion of viral RNA in certain subgroups. Identifying these subgroups is crucial to know how prioritize preventive strategies in elderly.

## Introduction

Coronavirus disease 2019 was first reported in Wuhan, China in December 2019 (1). It spread rapidly to more than 216 countries and it was declared to be pandemic by The World Health Organization (WHO) on March 11, 2020 (2). Until April 26, 2023, 764,312,098 cases of COVID-19 and 6,931,726 related deaths have been recorded worldwide (3).

The disease has drastically expanded in Tunisia. Until the 26^th^ of April 2023, 1,152,483 COVID-19 have been reported in Tunisia, and 29,378 have died (4). COVID-19 symptoms range from mild (fever and respiratory symptoms) to severe (pneumonia, severe acute respiratory syndrome, kidney failure and thrombotic complications) with a mortality rate of 3.4% globally at the beginning of the pandemic (5).

Several studies had demonstrated that old age is a potential predictor of mortality and severe forms of COVID-19 (6, 7). Age-related immune system defects resulting in a weak immunity response support this higher severity in elderly (8).

However, as regards viral clearance, the relationship between age and time to negative conversion remains controversial. The relationship was established in hospitalized patients and severe forms of COVID-19 (8, 9). Whereas, among patients with mild forms of COVID-19, age was not independently associated with prolonged time to viral conversion according to several studies (10–14). However, identifying subgroups at risk of delayed RNA negative conversion among elderly with mild forms of COVID-19 is crucial to establish preventive strategies among them (15).

To the best of our knowledge, there are no studies that have identified individuals at risk of prolonged time to viral conversion among elderly with non-severe forms of COVID-19.

Studying the relationship between age and SARS-CoV-2 viral clearance duration among non-severe COVID-19 patients is of a great importance and can have significant public health implications. It might influence clinical decisions on isolation, surveillance strategies and vaccination priority.

The aim of this study was to investigate the relationship between age and time to negative conversion of SARS-CoV-2 in patients with asymptomatic and mild forms of COVID-19.

## Methods

### Study design

This is a retrospective cohort study that included all patients with non-severe forms of COVID-19 in the national containment center of Monastir from March 2020 to July 29, 2020.

### Setting

The national containment center was dedicated for asymptomatic and mild COVID-19 cases. The government had allocated this center, during the first wave of COVID-19, for cases who were unable to be self-isolated at home in order to avoid the spread of the epidemic. During the study period, this center was the unique containment center in Tunisia and received asymptomatic and mild COVID-19 cases from all Tunisian governorates.

### Case definition

A confirmed COVID-19 case was defined as any individual, symptomatic or not, with laboratory confirmation of SARS-CoV-2 infection using real-time reverse transcriptase–polymerase chain reaction (RT-PCR) (16).

### Viral clearance monitoring and criteria

#### Enrollment process

All patients diagnosed with asymptomatic or mild forms of COVID-19 at the time of admission to the national containment center were initially included in the study. Patients with secondary aggravation to moderate or severe forms were then excluded.

Exclusion criteria were moderate and severe cases. Moderate COVID-19 cases are patients who presented with lower respiratory tract disease on clinical assessment or imaging and had an oxygen saturation measured by pulse oximetry (SpO2) ≥94%. Severe cases requiring hospitalization or intensive care unit (ICU) admission for COVID-19 complications such as respiratory failure, septic shock and/or multi-organ dysfunction were also excluded from the study (17). Thus, 289 patients were included for analysis.

#### Exposure

Patients included in the study were subdivided into two cohorts: (under 60 years) and (over 60 years) and were followed up to viral clearance. Both groups received only symptomatic treatment when indicated.

#### Follow up

The follow-up involved a weekly RT-PCR testing for SARS-CoV-2 to check for viral clearance. If the first SARS-CoV-2 test was still positive, then the second test was done 7 days later. Those individuals with two consecutive negative RT-PCR test results within 24 h were then considered virus-free and were discharged from the containment center.

## Data collection and definitions

Time to viral clearance was defined by the number of days from the first positive SARS-CoV-2 RT-PCR test results to the persistent first negative of two consecutive negative RT-PCR findings (8, 12, 18, 19).

Explanatory variables were collected from medical records and through phone calls during the containment period. The following data were extracted: Gender, past medical history, body mass index (BMI), symptoms, respect of isolation measures during the containment period and complications.

Past medical history was defined as having one or more of the following diseases (diabetes, hypertension, cardiovascular disease, dyslipidemia, respiratory disease, cancer, …).

Symptomatic forms were defined as positive cases with one or more known symptoms of COVID-19, including but not limited to cough, fever, headache, muscle pain, shortness of breath, anosmia and ageusia. Mild cases included positive patients for SARS-CoV-2 who have one or more of the known symptoms of COVID-19 but without a shortness of breath, dyspnea or abnormalities on chest imaging (17).

### Laboratory confirmation of SARS-CoV-2 infection

Detection of SARS-CoV-2 in nasopharyngeal (NP) specimens was performed at a reference laboratory. RNA was extracted from NP swabs using the GXT NA extraction kit (HainlifescienceGmbh, Nehren, Germany) or the QIAamp Viral RNA Mini kit (Qiagen®, Courtaboeuf, France) following the manufacturers’ instructions. With this assay, a positive COVID-19 result is determined when both targets (N and Orf1ab) reach a defined cycle threshold (Ct) of less than 40 (20). PCR testing was performed for patients with clinical symptoms and for asymptomatic ones identified through contact tracing tested within 24–48 h.

During our study, SARS-CoV-2 infection was only confirmed by testing respiratory specimens based on RT-PCR assays from different agreed institutions, including the University of Monastir laboratory.

### Statistical analysis

Data were entered and analyzed using IBM SPSS Statistics version 21.0 software. Continuous variables were expressed as median values with interquartile intervals. The categorical data were described in terms of percentages and frequencies.

Kaplan–Meier curves were used to estimate the cumulative RNA negativity rate and to compare time to negative conversion between elderly and young people in different strata. Cox regression analyses were applied to analyze the relationship between age and time to negative conversion. The Cox proportional hazards assumption was assessed graphically using Kaplan–Meier curves which revealed no violations of the proportional hazards assumption.

Model 1 examined the association without adjusting for confounding variables. Model 2 adjusted to take into account the most common confounding biases, which include gender, comorbidities, and symptoms. Cox regression was then performed with interaction terms: (between age and gender) and (between age and comorbidities) in models 3, 4, and 5.

Subgroup analysis of Cox regression was conducted to assess the relationship between age and time to negative conversion by, gender, comorbidities, symptoms … *p* values of <0.05 or interaction *p* values of <0.10 were considered statistically significant.

### Ethical considerations

The study was conducted in accordance with the ethical principles of the Declaration of Helsinki. The school of medicine of Monastir approved the study protocol.

## Results

### Clinical features of the study population

The study included 289 patients with positive RT-PCR for SARS-Cov2 (Median age 40 years (IQR; 28–54 years), with a sex ratio of 1.02). The young group included 245 individuals (Median age 35 years (IQR; 27–48 years), with a sex ratio of 0.99) whereas the elderly group (≥ 60 years) included 44 individuals (Median age 64 years (IQR; 61–72 years), with a sex ratio of 1.2).

There was no statistically significant difference between the two groups according to the gender, BMI, smoking status, symptoms and respect of isolation measures. However, patients in the elderly group were more likely to have past medical history especially hypertension and diabetes. The clinical features of the two groups were described in Table 1.

**Table 1:** General characteristics of 289 patients with confirmed SARS-Cov-2 infection.

	Young group (*n* = 245)	Elderly group (*n* = 44)	*p*
Age, years, median [IQR]	35 [27–48]	64 [61–72]	**< 10**^**−3**^
Gender, male, *n* (%)	122 (49.8)	24 (54.4)	0.56
Current smoker, *n* (%)	21 (10.4)	1 (3.4)	0.23
Past medical history	50 (22.8)	27 (62.8)	**< 10**^**−3**^
Hypertension, *n* (%)	19 (8.6)	19 (44.2)	**< 10**^**−3**^
Diabetes, *n* (%)	27 (12.2)	15 (34.1)	**< 10**^**−3**^
Respiratory disease, *n* (%)	12 (5.4)	3 (7.1)	0.66
BMI, kg/m^2^, median [IQR]	25.9 [22.8–28.3]	27.9 [24.1–29.4]	0.49
Symptomatic patients, *n* (%)	66 (32.0)	13 (37.1)	0.55
Respect of isolation measures, *n* (%)	168 (91.3)	18 (81.8)	0.15

IQR, iner-quartile range. Bold values means *p* < 0.05.

Median time to viral clearance was not statistically different between the two groups 20 [16–32] *Vs* 21 [17–33] (*p* = 0.057) among young and elderly, respectively.

### Subgroup analyses of time to viral clearance according to age (Young group vs. older group)

Table 2 shows that Age ≥ 60 was an associated factor to prolonged negative conversion of viral RNA for male sex (HR:1.9; 95% CI: 1.2–3.03) and patients with chronic conditions (HR:1.68; 95% CI: 1.02–2.75) especially diabetics (HR:2.06; 95% CI: 1.01–4.21). This association increased to (HR:2.30; 95% CI: 1.13–4.66) when male sex and comorbidities were concomitantly present and rose to (HR: 2.63; 95% CI: 1.02–6.80) for men with diabetes.

**Table 2:** Comparison of time to viral clearance in days according to general characteristics among young and elderly group.

		Young group (*n* = 245) Median [IQR]	Elderly group (*n* = 44) Median [IQR]	*p*	HR 95%CI
**Crude analysis**					
		20 [16–32]	21 [17–33]	0.057	1.38 [0.99–1.93]
**Stratified analysis**					
**One factor**					
Gender	Female (146)	24 [17–36]	20 [17–29]	0.34	0.79 [0.49–1.28]
	Male (143)	19 [16–28]	28 [17.5–43.5]	**0.004**	**1.90 [1.20–3.07]**
Current smoker	No (209)	21 [16–34]	20 [17–32.75]	0.70	1.08 [0.72–1.62]
	Yes (22)	20 [17–26.5]	42	0.25	3.24 [0.42–25.02]
Chronic condition	No (185)	21 [17–33.5]	24 [16.25–37.5]	0.67	1.11 [0.66–1.86]
	Yes (77)	18 [15–29.25]	22 [17–33]	**0.02**	**1.68 [1.02–2.75]**
Hypertension	No (227)	21 [17–33]	20 [16–32.75]	0.45	1.18 [0.76–1.82]
	Yes (38)	16 [15–28]	22 [17–33]	0.11	1.70 [0.88–3.30]
Diabetes	No (224)	21 [17–34]	20 [17–33]	0.43	1.17 [0.78–1.74]
	Yes (42)	18 [15–24]	22 [16–44]	**0.03**	**2.06 [1.01–4.21]**
Respiratory disease	No (248)	21 [16–32.5]	20 [17–33]	0.10	1.32 [0.92–1.89]
	Yes (15)	17.5 [15–33.75]	25 [22.5–31.5]	0.12	1.32 [0.40–5.42]
Overweight	No (65)	21 [17–36]	26.5 [18.5–43]	0.60	1.24 [0.53–2.90]
	Yes (85)	21 [17–34]	20 [15.75–28]	0.25	0.69 [0.37–1.30]
Symptomatic patients	No (162)	21 [16–31.5]	21 [16.75–31.25]	0.39	1.22 [0.77–1.94]
	Yes (79)	23.5 [18–36]	20 [17.5–36]	0.79	1.08 [0.58–2.01]
**Associated factors**					
Male sex and chronic condition	No (232)	21 [17–33.5]	20 [17–31]	0.82	0.95 [0.63–1.43]
	Yes (42)	17.5 [15–33.25]	28.5 [19.25–47.75]	**0.01**	**2.30 [1.13–4.66]**
Male sex and diabetes	No (231)	21 [16–33]	20 [17–32]	0.55	1.11 [0.78–1.58]
	Yes (22)	17.5 [15–31]	27 [18.25–51]	**0.03**	**2.63 [1.02–6.80]**

IQR, iner-quartile range, HR, hazard ratio, 95%CI: 95% confidence interval. Bold values means *p* < 0.05.

**Figure 1:**
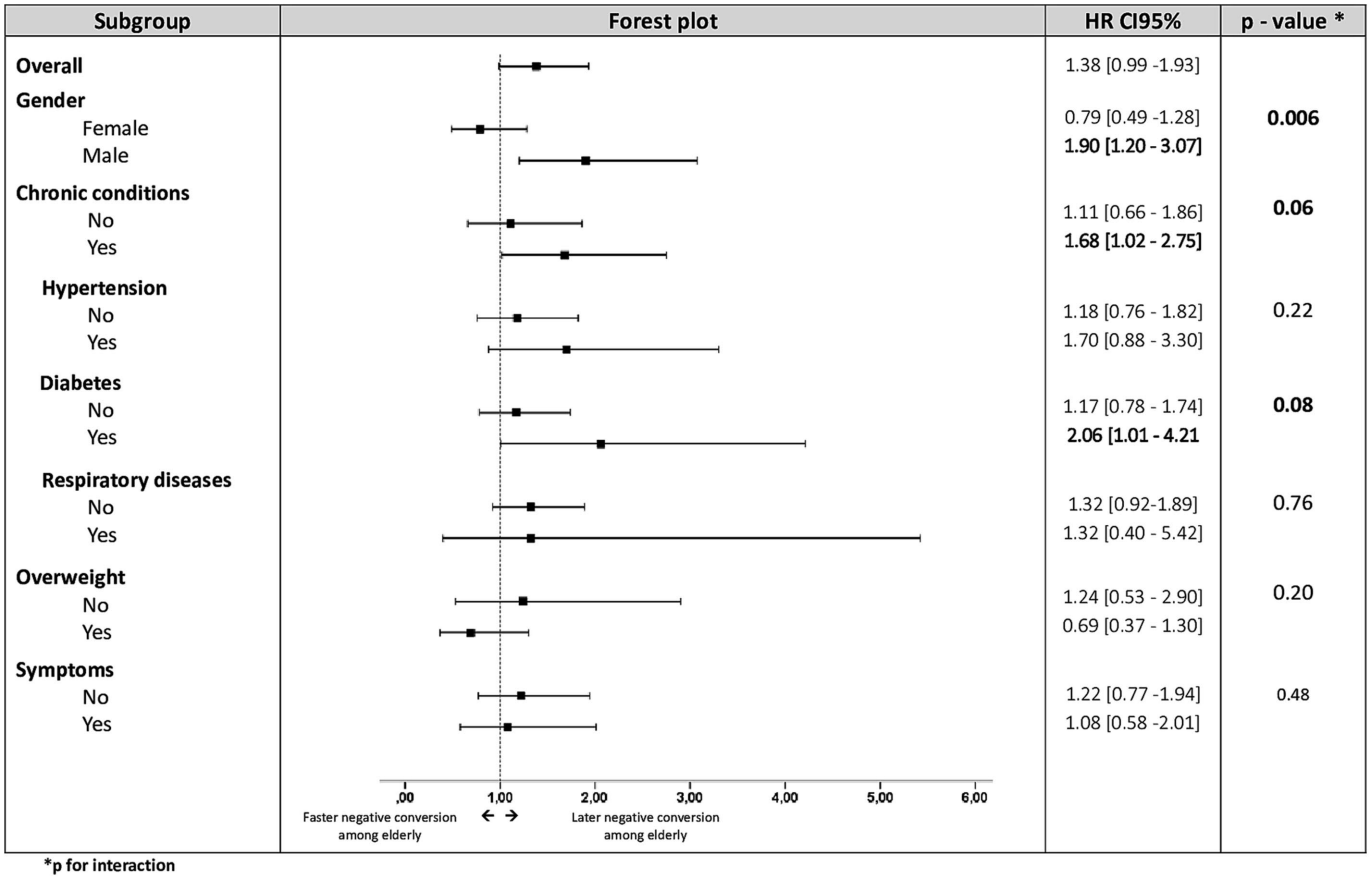
Comparison of time to negative conversion between young and elderly by subgroup.

**Figure 2:**
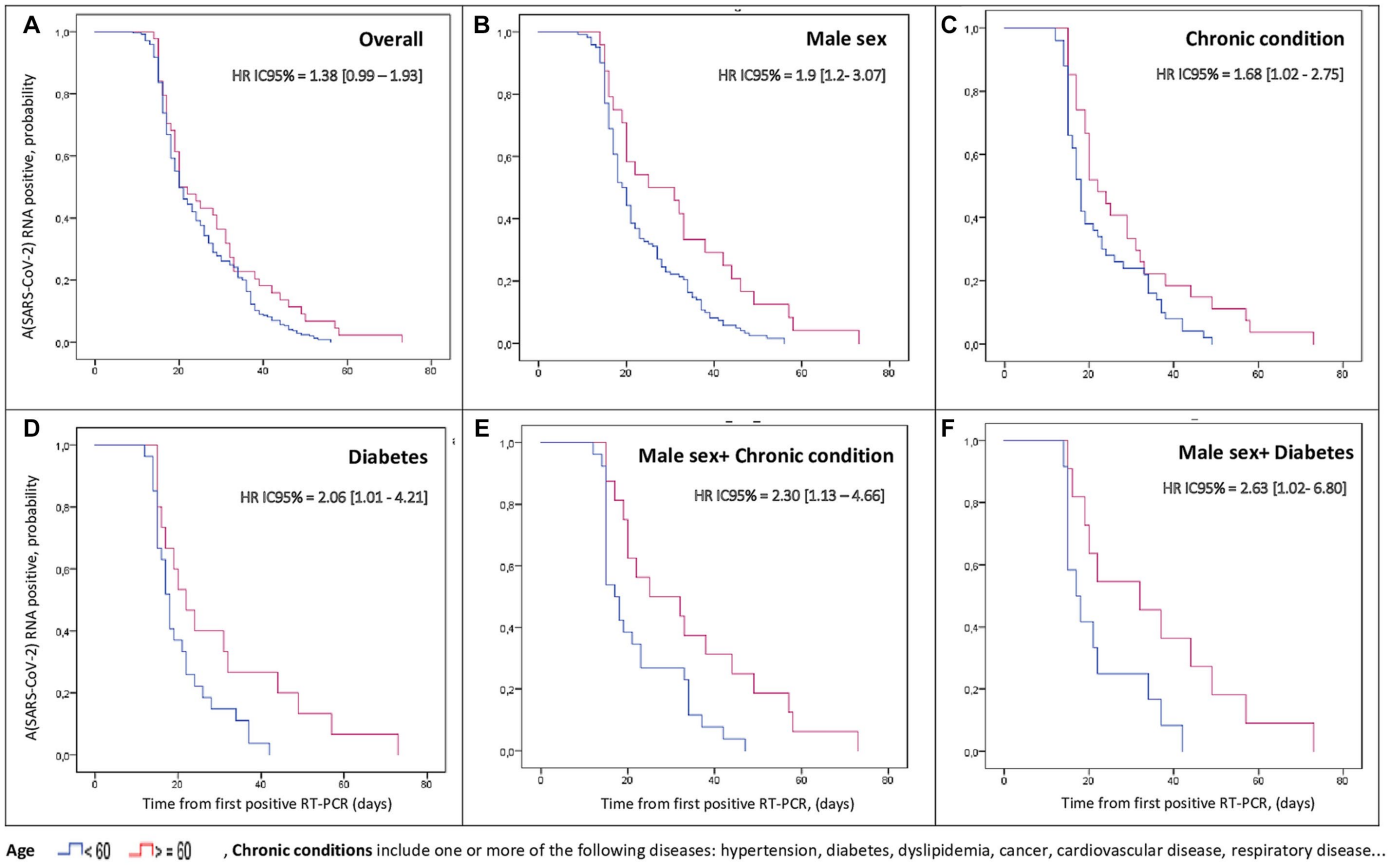
Stratified cumulative percentage of patients with positive SARS-CoV-2 RNA by time after illness onset.

Figures 1, 2 shows the Comparison of time to negative conversion between young and elderly by subgroup.

### Multivariate analysis

After adjusting for gender, comorbidities and symptoms and testing for interaction, cox regression analysis revealed a significantly delayed negative conversion in the group of symptomatic patients (Models 4 and 5). Significant interaction was observed between gender and age (Models 3 and 5) and between age and chronic conditions (Model 4) (Table 3).

**Table 3:** Factors associated with delayed negative conversion of viral RNA (Multivariate analysis).

	Model 1			Model 2			Model 3			Model 4			Model 5		
	*p*	HR	95% CI	*p*	HR	95% CI	*p*	HR	95% CI	*p*	HR	95% CI	*p*	HR	95% CI
Age ≥ 60	0.057	1.38	[0.99–1.93]	0.21	1.30	[0.85–1.99]	0.28	0.73	[0.41–1.29]	0.40	0.76	[0.41–1.42]	0.06	0.51	[0.25–1.02]
Male gender			**-**	0.56	1.08	[0.82–1.42]	0.74	0.95	[0.71–1.27]	0.61	1.07	[0.81–1.40]	0.77	0.95	[0.72–1.28]
Chronic condition			**-**	0.24	0.82	[0.59–1.40]	0.17	0.80	[0.58–1.10]	0.05	0.71	[0.50–1.01]	0.06	0.72	[0.51–1.01]
Symptoms			**-**	0.08	1.27	[0.96–1.69]	0.08	1.28	[0.96–1.70]	**0.04**	**1.34**	**[1.01–1.79]**	**0.04**	**1.33**	**[1.01–1.77]**
(Age ≥ 60) *Male gender			**-**			**-**	**0.01**	**0.37**	**[0.17–0.80]**			**-**	**0.02**	**0.42**	**[0.19–0.91]**
(Age ≥ 60) *Chronic condition			**-**			**-**			-	**0.04**	**0.43**	**[0.19–0.98]**	0.12	0.52	[0.22–1.20]

Model 1: without adjusting for confounding variables. Model 2: adjusted to take into account the most common confounding biases, which include gender, comorbidities, and symptoms. Model 3: adjusted for gender, comorbidities, and symptoms and tested for interaction between age and gender. Model 4: adjusted for gender, comorbidities, and symptoms and tested for interaction between age and chronic conditions. Model 5: adjusted for gender, comorbidities, and symptoms and tested for interaction (age*gender) and (age*chronic conditions). Bold values means *p* < 0.05.

## Discussion

To the best of our knowledge, there has been little investigations focusing on SARS-CoV-2 negative conversion duration among elderly compared with young. According to the findings of our study: Age was not found to be an independent predictor of delayed viral clearance but stratified analysis showed that age ≥ 60 was significantly associated with longer viral clearance duration for male-sex and for patients with comorbidities. This association was stronger when there was a concomitant presence of these two factors. Cox regression analysis revealed significant interactions between (gender and age) and between (age and chronic conditions). Thus, we can conclude from these results that sex and comorbidities would be effect modifiers of the relationship between age and time to SARS-CoV-2 negative conversion.

Our two groups were comparable according to the main demographic and clinical features: gender, BMI, smoking status, respect of isolation measures and the proportion of symptomatic patients. In line with our findings, no statistically significant difference was found between the proportion of symptomatic patients in the two groups according Sherwal et al. (21). Whereas, Mori et al. (1) reported a significantly higher proportion of symptomatic cases among elderly.

Regarding comorbidities, patients in the elderly group were more likely to have past medical history especially hypertension and diabetes which was a common finding in most studies dealing with the same topic (1, 22).

The median time to viral clearance observed in our study was 20 days [IQR: 16–33 days, similar to a previous study (6)]. It was 20 days (IQR; 16–32 days) for patients under 60 years, and 21 days (IQR; 17–33 days) for patients ≥60 years. Another study demonstrated a shorter median duration of time to negative conversion of 16.5 (IQR:11.8–20) days in elderly (1). However, a Japanese study reported a longer median duration of 26 ± 13.5 days (23).

Several reports have shown that the older age was associated to delayed RT-PCR conversion (1, 8, 9) and higher viral load (16, 17). These findings were explained by the fact that due to immunosenescence and inflammation in some older adults, the SARS-CoV-2 escape the immune surveillance and leads to prolonged viral shedding (24). Immunosenescence is associated with the incapacity to provide protective humoral and cellular immune responses against a pathogen (25). As for inflammation aging play a role in increasing systemic inflammation leading to hyperactive but less protective immune system (26).

However, similar to our study, age was not found to be an independent predictor of delayed viral clearance (15, 18). The discrepancy between studies might be attributed to the differences in disease severity of the study population. Our results could be attributed to the fact that we focused only on patient with non-severe forms of COVID-19. Old age incrimination in prolonged time to negative conversion was demonstrated for specific subgroups in our study: The finding of delayed viral clearance in the elderly group with comorbidities comparatively to the young group with comorbidities (HR 1.68) could be explained by a synergy between age and co-morbidities especially diabetes in prolonging the duration of the disease. It was found, according Tadic et al., that serum levels of inflammation biomarkers were significantly higher in patients with diabetes compared with those without, indicating that these patients are predisposed to an hyper-inflammatory state (27). This hyper-inflammatory state, which is more common among elderly, might increase susceptibility for prolonged negative conversion in aged COVID-19 patients. That is why; special consideration should be given to elderly with underlying disease especially those with diabetes.

This study also suggests that for men, compared to young group, those with an age above 60 years have a prolonged time to negative conversion (HR 1.91). In addition to age related changes, past studies have shown that sex has an effect on infections outcome and has been associated with differences in immune responses (28). Males appear to be more susceptible to viral infections and develop lower responses to various types of vaccination than females (29). This might explain SARS-CoV-2 delayed viral clearance among males and more significantly among elderly men.

The concomitant presence of male sex and comorbidities increased the association between old age and delayed viral clearance (HR 2.30). The implication of comorbidities and male sex in viral clearance delay was reported in many studies (18). Cox regression analysis showed that being symptomatic was a determinant factor of prolonged negative RNA conversion similar to a previous study (30). Indeed, symptomatology such as fever might be a response to the release of inflammatory mediators such as cytokine (31). These inflammatory mediators are responsible of tissue damaging and organ dysfunction suggesting that SARS-CoV-2 RNA negative conversion could be prolonged in symptomatic patients (32). Therefore, COVID-19 patients with symptoms should seek special medical care to avoid delayed negativation and extend treatment time.

Some limitations should be considered in our study. First, we detected the existence of viral SARS-CoV-2 RNA only in nasal swabs and not patients’ excretions like urine and fecal specimens. Second, we had only qualitative results of RNA detection and not viral loads dynamic profiles. Third, our study did not include all variants of SARS-COV-2 that is why further studies targeting Delta an Omicron variant should be recommended.

To the best of our knowledge, this is the first study that focuses on risk levels of prolonged time to negative conversion in elderly people comparatively to young people. Our results have relevant public health implications and would help guide prevention strategies for old people.

## Conclusion

Our study highlighted that old age is associated with prolonged negative conversion of viral RNA in specific subgroup patients. The identification of these subgroups is crucial to knowing how to prioritize preventive strategies in elderly. It may help establishing risk levels of prolonged viral clearance for aged patients with COVID-19 in order to guide decisions on isolation, surveillance strategies and vaccination among this vulnerable category. These findings would be very useful even in future epidemics as emerging viral diseases have become increasingly common.

## Data availability statement

The raw data supporting the conclusions of this article will be made available by the authors, without undue reservation.

## Ethics statement

The studies involving humans were approved by the ethics committee of Faculty of medicine of Monastir. The studies were conducted in accordance with the local legislation and institutional requirements. The participants provided their written informed consent to participate in this study.

## Author contributions

IZ: Conceptualization, Formal analysis, Methodology, Writing – original draft. AB, IB, RK, CL, SM, and MM: Formal analysis, Writing – review & editing. CB, IC, WD, MK, MF, HA, and IJ: Formal analysis, Writing – original draft. AM and DH: Data collection and Writing – original draft. All authors contributed to the article and approved the submitted version.
